# Mechanical strain-mediated reduction in RANKL expression is associated with RUNX2 and BRD2

**DOI:** 10.1016/j.gene.2020.100027

**Published:** 2020-01-16

**Authors:** Gabriel L. Galea, Christopher R. Paradise, Lee B. Meakin, Emily T. Camilleri, Hanna Taipaleenmaki, Gary S. Stein, Lance E. Lanyon, Joanna S. Price, Andre J. van Wijnen, Amel Dudakovic

**Affiliations:** a Department of Orthopedic Surgery, Mayo Clinic, Rochester, MN, USA; b Developmental Biology and Cancer, UCL GOS Institute of Child Health, London, UK; c Comparative Bioveterinary Sciences, Royal Veterinary College, London, UK; d Mayo Clinic Graduate School of Biomedical Sciences, Mayo Clinic, Rochester, MN, USA; e Center for Regenerative Medicine, Mayo Clinic, Rochester, MN, USA; f School of Veterinary Sciences, University of Bristol, Bristol, UK; g Molecular Skeletal Biology Laboratory, Department of Trauma, Hand and Reconstructive Surgery, University Medical Center Hamburg-Eppendorf, Hamburg, Germany; h Department of Biochemistry, University of Vermont College of Medicine, Burlington, VT, USA; i Department of Biochemistry and Molecular Biology, Mayo Clinic, Rochester, MN, USA

**Keywords:** RANKL/TNFSF11, receptor activator of nuclear factor-κB ligand, SOST, Sclerostin, RUNX2, Runt-related transcription factor 2, BRD2, Bromodomain-containing protein 2, OPG, Osteoprotegerin/tumour necrosis factor receptor superfamily member 11B, PGE2, Prostaglandin E2, Ki-67, Antigen KI-67, HPRT, Hypoxanthine Phosphoribosyltransferase 1, GAPDH, Glyceraldehyde 3-Phosphate Dehydrogenase, β2MG, Beta-2-Microglobulin, ALP, Alkaline phosphatase, HDAC, Histone deacetylase, sh, Short hairpin, PCR, polymerase chain reaction, RT-qPCR, Quantitative reverse transcription polymerase chain reaction, CO_2_, Carbon Dioxide, DMEM, Dulbecco's Modified Eagle Medium, FCS, Fetal calf serum, IU, International unit, AzadC, 5-Aza-2′-deoxycytidine, ActD, Actinomycin D, eGFP, enhanced green fluorescent protein, FACS, Fluorescence-activated cell sorting, PBS, Phosphate-Buffered Saline, RNA, Ribonucleic Acid, DNA, Deoxyribonucleic Acid, DAPI, 4′,6-diamidino-2-phenylindole, IgG, Immunoglobulin G, ChIP, Chromatin immunoprecipitation, RUNX2, Mechanical strain, Epigenetics, BRD2, Sclerostin, Receptor activator of nuclear factor-κB ligand

## Abstract

Mechanical loading-related strains trigger bone formation by osteoblasts while suppressing resorption by osteoclasts, uncoupling the processes of formation and resorption. Osteocytes may orchestrate this process in part by secreting sclerostin (SOST), which inhibits osteoblasts, and expressing receptor activator of nuclear factor-κB ligand (RANKL/TNFSF11) which recruits osteoclasts. Both SOST and RANKL are targets of the master osteoblastic transcription factor RUNX2. Subjecting human osteoblastic Saos-2 cells to strain by four point bending down-regulates their expression of SOST and RANKL without altering RUNX2 expression. RUNX2 knockdown increases basal SOST expression, but does not alter SOST down-regulation following strain. Conversely, RUNX2 knockdown does not alter basal RANKL expression, but prevents its down-regulation by strain. Chromatin immunoprecipitation revealed RUNX2 occupies a region of the RANKL promoter containing a consensus RUNX2 binding site and its occupancy of this site decreases following strain. The expression of epigenetic acetyl and methyl writers and readers was quantified by RT-qPCR to investigate potential epigenetic bases for this change. Strain and RUNX2 knockdown both down-regulate expression of the bromodomain acetyl reader BRD2. BRD2 and RUNX2 co-immunoprecipitate, suggesting interaction within regulatory complexes, and BRD2 was confirmed to interact with the RUNX2 promoter. BRD2 also occupies the RANKL promoter and its occupancy was reduced following exposure to strain. Thus, RUNX2 may contribute to bone remodeling by suppressing basal SOST expression, while facilitating the acute strain-induced down-regulation of RANKL through a mechanosensitive epigenetic loop involving BRD2.

## Highlights

• Mechanical strain reduces SOST and RANKL expression.
• RUNX2 knockdown increases SOST levels.
• RUNX2 facilitates strain-mediated RANKL suppression.
• BRD2 expression is modulated by strain and RUNX2.
• Strain alters BRD2 and RUNX2 occupancy at the RANKL promoter.

## 1 Introduction

Mechanical strain caused by load-bearing informs the activity of osteoblasts which form new bone and osteoclasts which resorb surplus bone through mechanisms collectively referred to as functional adaptation. The opposing activities of these two cell types are normally balanced through coupling mechanisms involving direct and indirect signaling between members of their lineages (Sims and Martin, 2014). Key to this coupling is the secretion of receptor activator of nuclear factor κB ligand (RANKL) by osteoblastic lineage cells including terminally-differentiated osteocytes embedded within the bone matrix (Xiong et al., 2011; Kennedy et al., 2012). RANKL binds its receptor RANK on osteoclast precursors and promotes their differentiation. Its activity is opposed by the decoy receptor osteoprotegerin (OPG) also secreted by osteoblasts and osteocytes (Yasuda et al., 1998; Kennedy et al., 2012). OPG expression is increased by activation of the potently-osteogenic canonical Wnt signaling pathway, which is required for osteoblast differentiation in part by promoting expression of the osteoblastic master regulator RUNX2 (Holmen et al., 2005). Wnt signaling is antagonized by the osteocyte-specific secreted antagonist Sclerostin (SOST), which prevents excessive bone formation (van Bezooijen et al., 2004; Arbon et al., 2012).

It is widely accepted that osteocyte-derived signals including RANKL and sclerostin fine-tune (re)modeling (Kennedy et al., 2012; Moustafa et al., 2012). The mechanisms integral to functional adaptation-induced (re)modeling appear to fail with age, leading to excessive resorption relative to formation and a deterioration of bone mass and architecture characteristic of osteoporosis (Meakin et al., 2014; Galea et al., 2017). Elucidation of the cellular mechanisms involved has led to the clinical development of anti-RANKL antibody (Denosumab) and more recently and anti-sclerostin antibody (Romosozumab) (Compston et al., 2019). Expression of both SOST and RANKL is influenced by the transcription factor RUNX2 (Sevetson et al., 2004; Kitazawa et al., 2008; Byon et al., 2011). Furthermore, the expression of both SOST and RANKL is down-regulated in osteoblastic lineage cells subjected to mechanical strain (Kusumi et al., 2005; Robling et al., 2008).

Whereas acute SOST down-regulation following strain permits increased bone formation, RANKL down-regulation is expected to reduce the drive for resorption. Therefore, down-regulation of both these osteocyte-secreted products may contribute to the ability of mechanical loading to uncouple bone formation from resorption (Feher et al., 2010; Galea et al., 2011). In the functionally-adapted skeleton, basal secretion of these mediators establishes the osteogenic context on which acute (re)modeling stimuli are superimposed. For example, although osteoblastic cells are themselves strain-responsive, the presence of sclerostin in their environment reduces their strain-related increase in proliferation (Galea et al., 2013; Galea et al., 2015). Following exposure to acute episodes of increased strains beyond the habitual, down-regulation of sclerostin is spatially related to new bone formation (Moustafa et al., 2012).

To study the mechanisms by which strain down-regulates SOST, we have previously reported an in vitro model in which exposure of human female Saos-2 osteosarcoma cells to physiological levels of substrate strain by four point bending results in SOST down-regulation in a peak strain magnitude-dependent manner over a time course consistent with that observed following mechanical loading of rodent bones in vivo (Galea et al., 2011; Galea et al., 2013). Saos-2 cells have been extensively used to study SOST and RANKL regulation because of their mature osteoblastic phenotype and physiological expression of these genes (Sevetson et al., 2004; Galea et al., 2011; Arbon et al., 2012; Galea et al., 2013; Romagnoli et al., 2013; Prideaux et al., 2014). In our model, SOST down-regulation following strain involves estrogen receptor signaling (Galea et al., 2013) and the prostaglandin signaling pathway through PGE2/EP4 (Galea et al., 2011). In the absence of strain, PGE2 acts as a coupling agent, decreasing SOST expression but increasing RANKL (Tsutsumi et al., 2009; Blackwell et al., 2010; Galea et al., 2011; Genetos et al., 2011).

RANKL expression is also down-regulated in osteoblastic cells subjected to dynamic substrate strain (Rubin et al., 2000; Rubin et al., 2003; Fan et al., 2006; Rahnert et al., 2008; Kusumi et al., 2009), whereas simulated disuse in microgravity up-regulates RANKL (Rucci et al., 2007). In vivo disuse is commonly achieved through rodent tail suspension, which has been reported to increase RANKL (Tatsumi et al., 2007; Xiong et al., 2011; Moriishi et al., 2012). Studies investigating the effects of increased loading have, to our knowledge, not yet clearly demonstrated the context and bone compartments in which strain down-regulates RANKL expression in vivo. One report found that, in rats, exercise and vibration training sufficient to prevent bone loss caused by glucocorticoid treatment also blunted the glucocorticoid-induced increase in osteocyte RANKL expression (Pichler et al., 2013). In contrast, in vivo down-regulation of sclerostin by increased loading and its up-regulation following unloading have been extensively mapped (Robling et al., 2008; Blackwell et al., 2010; Gaudio et al., 2010; Arbon et al., 2012; Meakin et al., 2014).

The mechanisms by which mechanical stimulation reduces expression of both SOST and RANKL remain poorly delineated. Regulation of both these genes by the same transcription factor, RUNX2, led us to hypothesize that strain-mediated down-regulation of both SOST and RANKL involves RUNX2. In support of this hypothesis, mechanical stimulation activates RUNX2 in osteoblastic cells (Kanno et al., 2007; Arbon et al., 2012; Hassan et al., 2012), leading to increased expression of the osteoblastic differentiation marker osteocalcin (Arbon et al., 2012). However, whereas the role of RUNX2 in pre-osteoblasts has been extensively investigated, its actions in mature osteoblasts and osteocytes involved in functional adaptation to loading remain incompletely understood. Well-established functions of RUNX2 include its interactions with various epigenetic regulators such as histone deacetylase (HDAC) enzymes (Jensen et al., 2007; Lamour et al., 2007; Shimizu et al., 2010; McGee-Lawrence et al., 2011). HDACs turn off gene expression by removing acetylation groups recognized by acetyl reader enzymes including the bromodomain containing (BRD) proteins. Thus, we further hypothesized that RUNX2 could regulate SOST and RANKL expression by altering recruitment of epigenetic regulators to their promoters following strain. To investigate this we developed Saos-2 cells with stable transfection of short hairpin (sh)RUNX2 sequences or vector controls with which to study the effects of RUNX2 knockdown on SOST and RANKL regulation by mechanical strain.

## 2 Materials and methods

### 2.1 Cells, reagents and lentiviral transfection

Saos-2 cells were as previously described (Galea et al., 2011; Galea et al., 2013; Galea et al., 2014; Galea et al., 2015). Cells were maintained in phenol red-free DMEM containing 10% heat-inactivated FCS (PAA, Somerset, UK), 2 mM l-glutamine, 100 IU/ml penicillin and 100 IU/ml streptomycin (Invitrogen, Paisley, UK) (complete medium) in a 37 °C incubator at 5% CO_2_, 95% humidity as previously described (Galea et al., 2011; Galea et al., 2013). 5-Aza-2′-deoxycytidine (AzadC) was from Sigma-Aldrich (Dorset, UK) and dissolved in DMSO. For AzadC treatment Saos-2 cells were seeded in six-well plates at an initial density of 40,000 cells/cm^2^ in complete medium and allowed to settle overnight. Cells were then treated once with AzadC at a final concentration of 1 μM as previously reported to increase SOST expression by demethylating its promoter (Delgado-Calle et al., 2012) and harvested three days later. Actinomycin D (ActD) was also from Sigma-Aldrich (Dorset, UK) dissolved in ethanol and used at a final concentration of 2 μM 1 h prior to strain. shRUNX2 sequences were as previously reported (Pratap et al., 2008) cloned in pWTS1 vectors with enhanced green fluorescent protein (eGFP) for fluorescence-assisted cell sorting of shRUNX2- or pWTSL1 vector-transfected controls. shRUNX2 and pWTSL1 cells were treated identically throughout. For transfection, 20,000 Saos-2 cells were seeded in each well of 6-well dishes and allowed to adhere for 24 h in 5 ml of complete medium. A 100 μl lentiviral titer of shRUNX2 or pWSTL1 vector control was then added to each dish together with 5 μl Polybrene™ (4 μg/ml). The following day cells received a complete medium change to wash away any excess viral particles and 3 days later the cells were sub-cultured. Cells were further sub-cultured two more times in order to wash any residual virus and ensure stable transfection before eGFP FACS sorting. A 100 μl aliquot of sorted cells was re-sorted to confirm sorting accuracy and showed >98% of sorted cells fell within the expected fluorescence window. The sorted cells were expanded and cryopreserved.

### 2.2 In vitro mechanical strain

Mechanical strain was generated by four point bending as previously described (Galea et al., 2011; Galea et al., 2013; Meakin et al., 2014). In brief, cells were seeded at an initial density of 40,000 cells/cm^2^ on custom-made plastic slides in 1 ml of complete medium, allowed to settle overnight, flooded with 5 ml/slide of complete medium and allowed to grow to over-confluence for 3 more days as this increases their SOST expression (Byon et al., 2011; Galea et al., 2013). Cells were then serum-deprived in 2% charcoal/dextran stripped FCS overnight before exposure to strain through a brief period of 600 cycles of four point bending of the strips with a peak strain of 3400 με on a Zwick/Roëll materials testing machine (Zwick Testing Machines Ltd., Leominster, UK) with strain rates on and off of ~24,000 με/s, dwell times on and off of 0.7 s and a frequency of 0.6 Hz.

### 2.3 Quantitative reverse transcription polymerase chain reaction (RT-qPCR)

RT-qPCR was performed as previously described using our previously reported primers for SOST and the housekeeping gene β2-microglobulin (Galea et al., 2011; Galea et al., 2013). Human RUNX2 primers were: Forward 5′-TGCCTAGGCGCATTTCAGGTGC-3′, Reverse 5′-CCTGAGGTGACTGGCGGGGT-3′ producing a 151 bp amplicon. RANKL primers were from the Harvard Primer Bank (Spandidos et al., 2010) ID 197927084b1. Agarose gel visualization was as previously described (Galea et al., 2011). Primer sequences used for the panel of epigenetic regulators in our custom PCR array have been previously described (Dudakovic et al., 2017). Each experiment involved 5 static and 5 strained slides pooled together to have enough RNA for down-stream processing.

### 2.4 Immunofluorescence and proliferation assay

Saos-2 Ki-67 immunofluorescence and proliferation assays were performed as previously described (Galea et al., 2013). Human RUNX2 antibodies and NorthernLights™-conjugated secondary antibodies were purchased from R&D Systems. For immunofluorescence, cells were permeabilized in 0.5% *v*/v Triton™ X-100 for 15 min, blocked for 1 h in 10% horse serum in PBS, and incubated with the appropriate primary antibody at a 1:200 dilution overnight. The next day, cells were washed 3 × 5 min in PBS with 0.5% v/v Triton™ X-100 before being incubated with a 1:200 dilution of the secondary antibody for 1 h at room temperature in the dark. Cells were then washed again 3 × 5 min in PBS and mounted in Fluoroshield DAPI (Sigma-Aldrich, Dorset, UK). Images were captured on a Leica DMRB microscope with an Olympus DP7.2 digital camera.

### 2.5 Alkaline phosphatase activity assays

Alkaline phosphatase (ALP) activity assays corrected to total protein were performed as previously-described (Meakin et al., 2014). Saos-2 cells were seeded at an initial density of 10,000 cells/cm^2^ in 24-well plates and cultured for 7 days in complete medium with half medium changes every 2 days. Cells were fixed in ice-cold methanol and alkaline phosphatase activity determined.

### 2.6 Chromatin immunoprecipitation (ChIP)

RUNX2 and BRD2 ChIP assays were performed as previously described (Dudakovic et al., 2013; Dudakovic et al., 2016). Saos-2 cells were strained or kept as static controls and their DNA was cross-linked with 1% formaldehyde at room temperature for 15 min. Each experiment involved 5 static and 5 strained slides but all the slides needed to be pooled to have enough DNA for down-stream processing such that each n of 1 represents an independent experiment. ChIP primers were as follows: RUNX2 P1 Forward 5′-TCAGCATTTGTATTCTATCCAAATCC-3′, Reverse 5′-TGGCATCCAGAAGGATATAGCTTTT-3′; RANKL Promoter site A Forward 5′-CAAAGGTGTCCTCTGCGTCT-3′, Reverse 5′-CTCTGTCACTGAAGGGCCTC-3′; RANKL Promoter site B Forward 5′-CCACCCAAAGTGCTGGGATT-3′, Reverse 5′-ACCTGCAATTCTTTGGTGGC-3′.

### 2.7 Western blotting

Western blotting was performed as previously described (Dudakovic et al., 2013) on immuno-precipitated ChIP lysates from Saos-2 cells subjected to strain or kept as static controls. RUNX2 and IgG antibodies are from Santa Cruz, and the BRD2 antibody is from Cell Signaling. Blots were visualized with an ECL^+^ detection kit using a FluorChem M CCD camera (Cell Biosciences).

## 3 Results

### 3.1 Mechanical strain down-regulates SOST and RANKL without altering RUNX2 expression

**Fig. 1:**
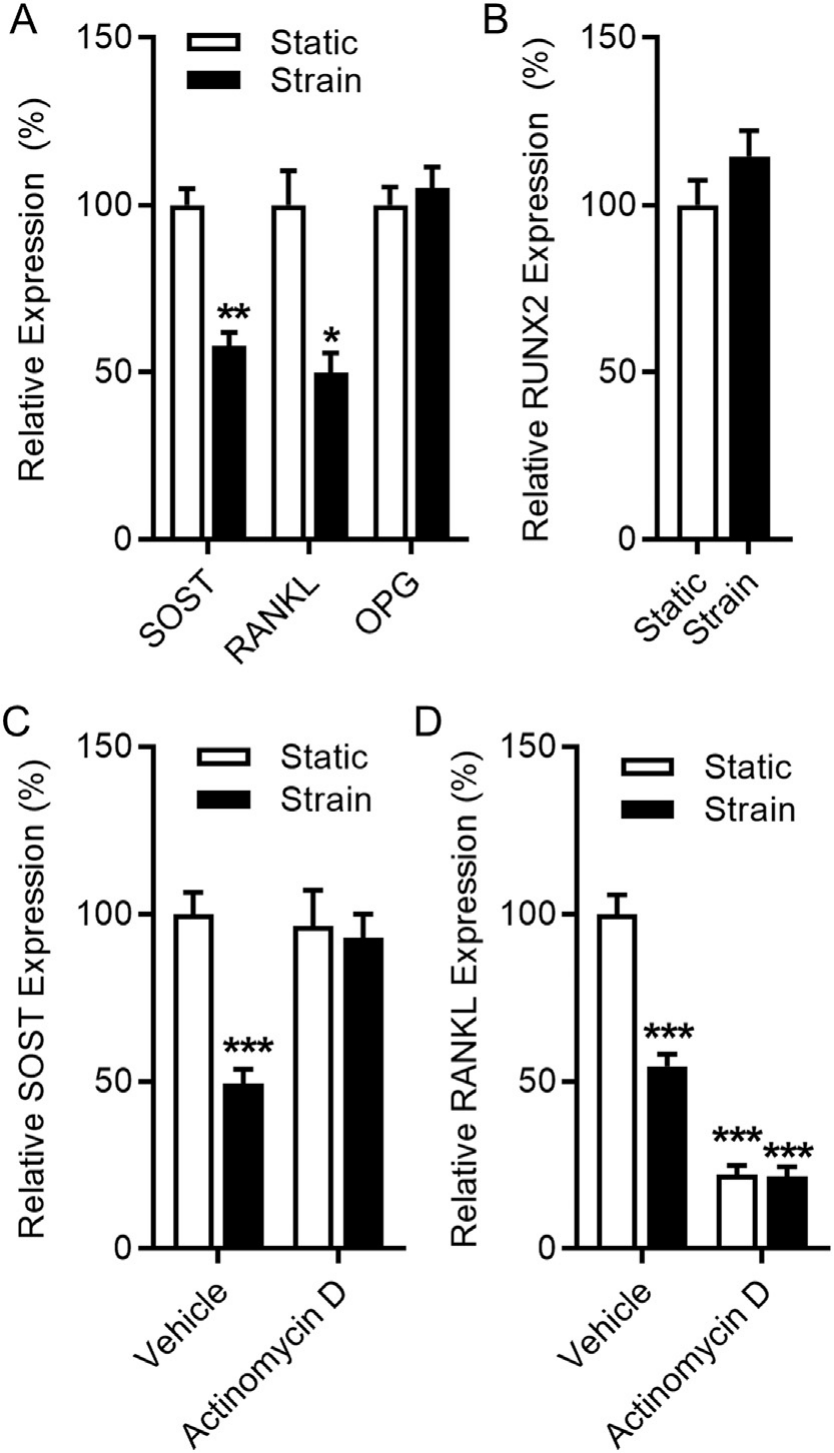
Mechanical strain alters gene expression in Saos-2 cells. Mechanical strain down-regulates SOST and RANKL (TNFSF11) expression, but does not alter OPG (TNFRSF11B) and RUNX2 expression in Saos-2 cells. RT-qPCR analysis of SOST, RANKL, OPG (A) and RUNX2 (B) subjected to strain for 8 h. RT-qPCR analysis of SOST (C) and RANKL (D) of cells pre-treated with 2 μM Actinomycin D or vehicle 1 h before exposure to strain for 8 h. Bars represent the mean ± SEM, *n* = 10–15. * *p* < .05, ** *p* < .01, *** *p* < .001 versus static controls.

Exposure of Saos-2 cells to strain down-regulates SOST and RANKL, but not OPG expression 8 h later relative to static controls (Fig. 1A). Strain had no significant effect on RUNX2 expression (Fig. 1B). Despite their similar temporal regulation by strain, differences in SOST and RANKL regulation are observed when new RNA synthesis is inhibited with ActD pre-treatment 1 h before strain. ActD treatment had no effect on SOST basal expression relative to the housekeeping gene but prevented its down-regulation by strain (Fig. 1C), whereas the same treatment reduced relative RANKL expression to a level lower than strain and the superimposition of strain had no further effect (Fig. 1D). Together, our data demonstrate that mechanical strain suppresses expression of SOST and RANKL in Saos-2 cells.

### 3.2 Characterization of shRUNX2 cells

**Fig. 2:**
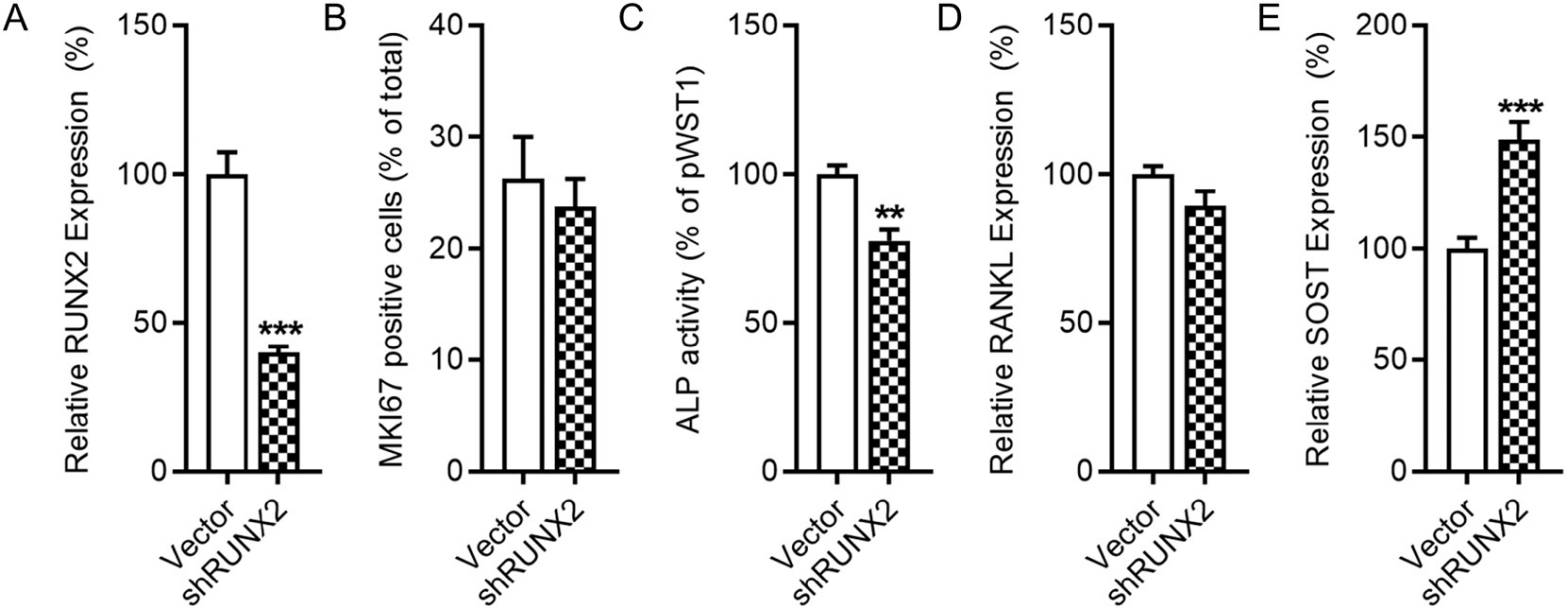
RUNX2 suppresses expression of SOST. Stable knockdown of RUNX2 in decreases alkaline phosphatase activity and increases SOST expression, but does not alter proliferation and RANKL levels in Saos-2 cells. RT-qPCR analysis of RUNX2 in vector control and shRUNX2 transfected cells (A). Percentage of Ki-67 positive cells (B) and alkaline phosphatase activity (ALP) (C) in vector control or shRUNX2 transfected cells. RT-qPCR analysis of RANKL (D) and SOST (E) in vector control or shRUNX2 transfected cells. Bars represent the mean ± SEM, n = 10–12 with the exception of B (*n* = 4). ** p < .01, *** p < .001 versus vector controls.

Transfection of Saos-2 cell with a lentiviral vector containing shRUNX2 and eGFP followed by FACS sorting permitted derivation of a stable cell line with greatly reduced RUNX2 expression relative to similarly transfected and sorted cells expressing the control pWST1 vector also expressing eGFP (Fig. 2A). RUNX2 knockdown did not alter basal proliferation as assessed with Ki-67 staining (Fig. 2B), but significantly reduced alkaline phosphatase (ALP) activity (Fig. 2C). RUNX2 knockdown did not alter basal RANKL expression (Fig. 2D), but it up-regulated SOST expression (Fig. 2E). These results demonstrate that RUNX2 knockdown increases SOST expression is Saos-2 cells.

### 3.3 RUNX2 suppresses basal SOST expression and mediates RANKL down-regulation by strain

**Fig. 3:**
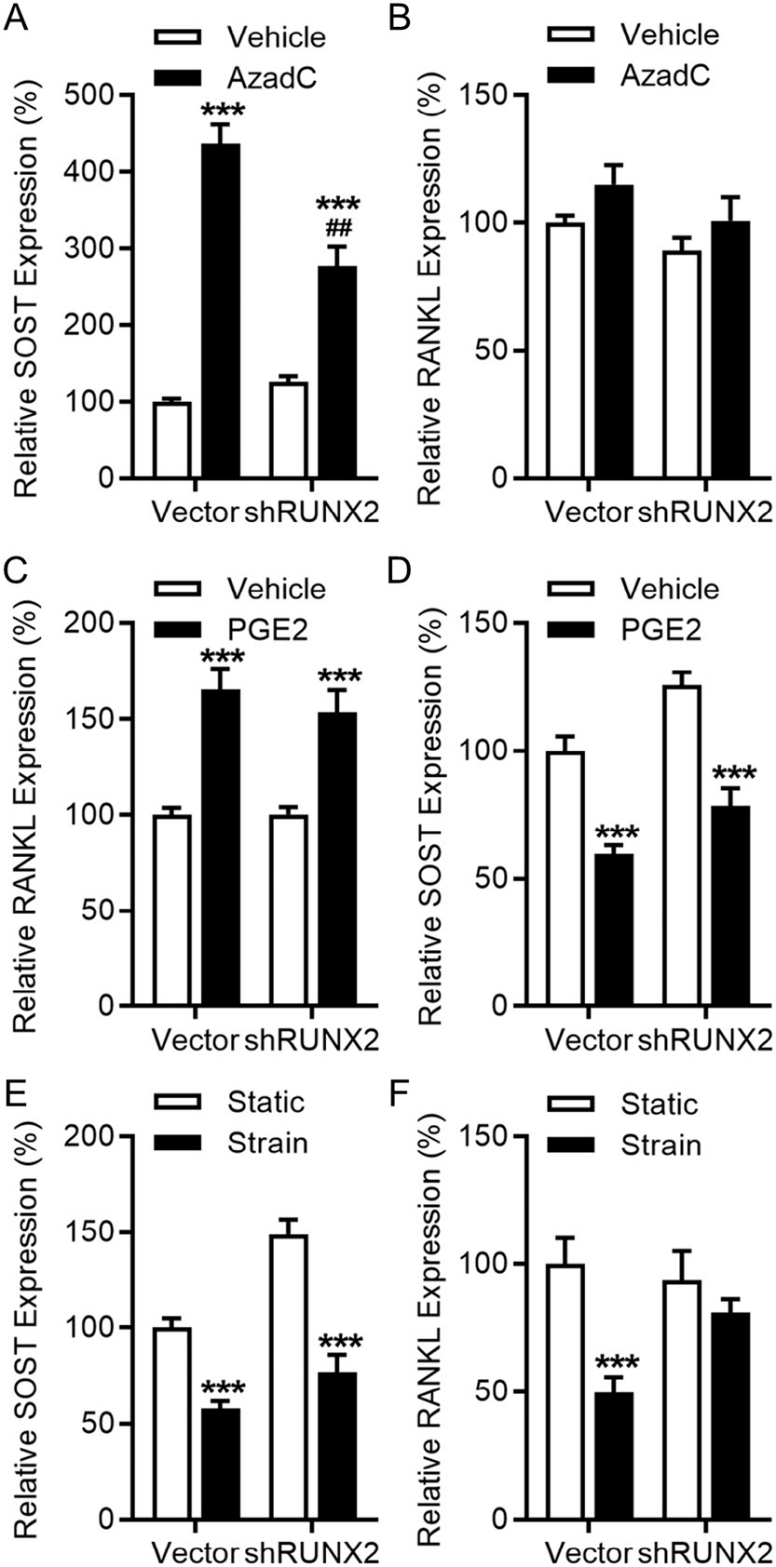
Strain-mediated suppression of SOST proceeds independently of RUNX2. RUNX2 influences basal SOST expression but not regulation of SOST by strain, whereas strain-related RANKL down-regulation but not basal expression are influenced by RUNX2. Cells were treated with 1 μM AzadC for 3 days and assessed for SOST (A) and RANKL (B) expression by RT-qPCR analysis (n = 10). Vector control or shRUNX2 cells were treated with vehicle or 0.5 μM PGE2 and harvested 24 h later to assess for RANKL (C) and SOST (D) expression by RT-qPCR analysis (n = 10). Cells were subjected to strain and harvested 8 h later to assess for SOST (E) and RANKL (F) expression by RT-qPCR analysis (*n* = 15). Bars represent the mean ± SEM. ** p < .01, *** p < .001 versus static controls of the same cell type, ^###^ p < .001 versus AzadC-treated pWTS1 cells.

RUNX2 may modulate SOST expression in Saos-2 cells by either activating (Sevetson et al., 2004) or suppressing (Byon et al., 2011) its promoter activity. Under our conditions, when SOST expression was increased with the DNA methylation inhibitor AzadC as previously reported (Delgado-Calle et al., 2012), cells lacking RUNX2 up-regulated SOST to a significantly lower extent than vector control cells (Fig. 3A). As neither RUNX2 knockdown nor AzadC had any significant effect on RANKL expression (Fig. 3B), we investigated the effect of the coupling agent PGE2 which is known to up-regulate RANKL. PGE2 up-regulated RANKL 8 h after treatment with no significant difference between shRUNX2 and vector control cells (Fig. 3C). Conversely, PGE2 down-regulated SOST expression in both shRUNX2 and vector control cells (Fig. 3D). Similarly, strain down-regulated SOST expression to a similar extent in vector control and shRUNX2 cells (Fig. 3E). However, strain was unable to down-regulate RANKL in cells lacking RUNX2 expression (Fig. 3F). Altogether, these results indicate that RUNX2 is involved in the strain-related down-regulation of RANKL but not SOST.

### 3.4 Strain reduces RUNX2 occupancy at the RANKL promoter

**Fig. 4:**
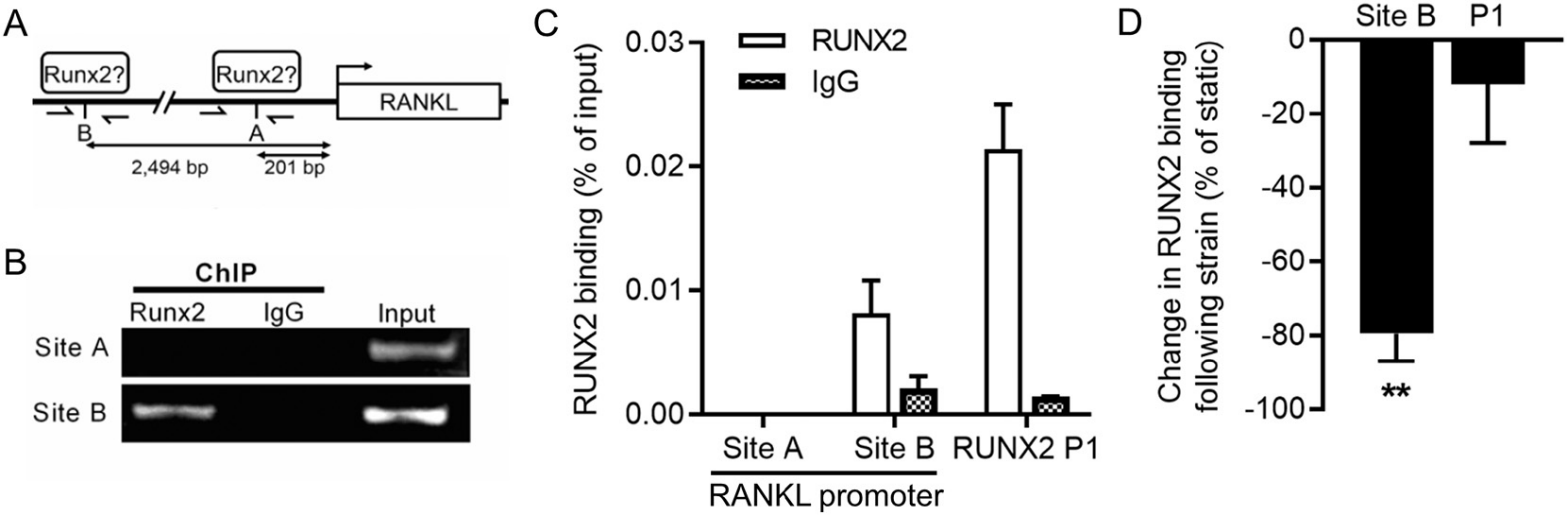
RUNX2 occupancy of the RANKL promoter decreases following strain. Schematic representation of the RANKL promoter that encompasses two RUNX2 recognition motifs (5′-ACCACA) denoted Site A (201 bp from the isoform 1 start site) and Site B (2,494 bp from the start site) in Saos-2 cells (A). Half-arrows indicate PCR amplicons amplified. Agarose gel images of PCR-amplified ChIPs with either RUNX2 antibody or IgG negative control antibody, and 1% input PCR positive control, using primer pairs specific for Site A or Site B in the RANKL promoter (B). PCR quantification of ChIPs with RUNX2 or IgG antibodies using RANKL promoter or the RUNX2 P1 promoter primers (ND = not detected) (C). Percent change in RUNX2 occupancy of the RANKL Site B and RUNX2 P1 promoter 8 h following strain (D). Bars represent the mean ± SEM, *n* = 3 representing three independent experiments. **p < .01 for the effect of strain.

To further delineate the RUNX2-dependant mechanisms by which strain down-regulates RANKL, we investigated RUNX2 binding to the RANKL promoter. An ACCAC putative RUNX2 binding site 201 bp from the isoform 1 start site had previously been suggested based on bioinformatic analysis of the RANKL promoter sequence in Saos-2 cells (Kitazawa et al., 2003). This site will be referred to as Site A. Additionally, a second RUNX2 consensus binding sequence was identified 2494 bp from the start site, denoted Site B (Fig. 4A). ChIP analysis revealed RUNX2 occupancy of Site B but not Site A in confluent Saos-2 cells (Fig. 4B&C). The RUNX2 P1 promoter, known to be robustly occupied in Saos-2 cells (van der Deen et al., 2012), was used as a positive control in ChIP assays. Strain significantly reduced RUNX2 occupancy at the RANKL promoter Site B without altering occupancy of the RUNX2 P1 promoter (Fig. 4D). In summary, these results demonstrate that RUNX2 occupancy at the RANKL promoter is reduced by strain.

### 3.5 BRD2 is a strain and RUNX2 target gene

**Fig. 5:**
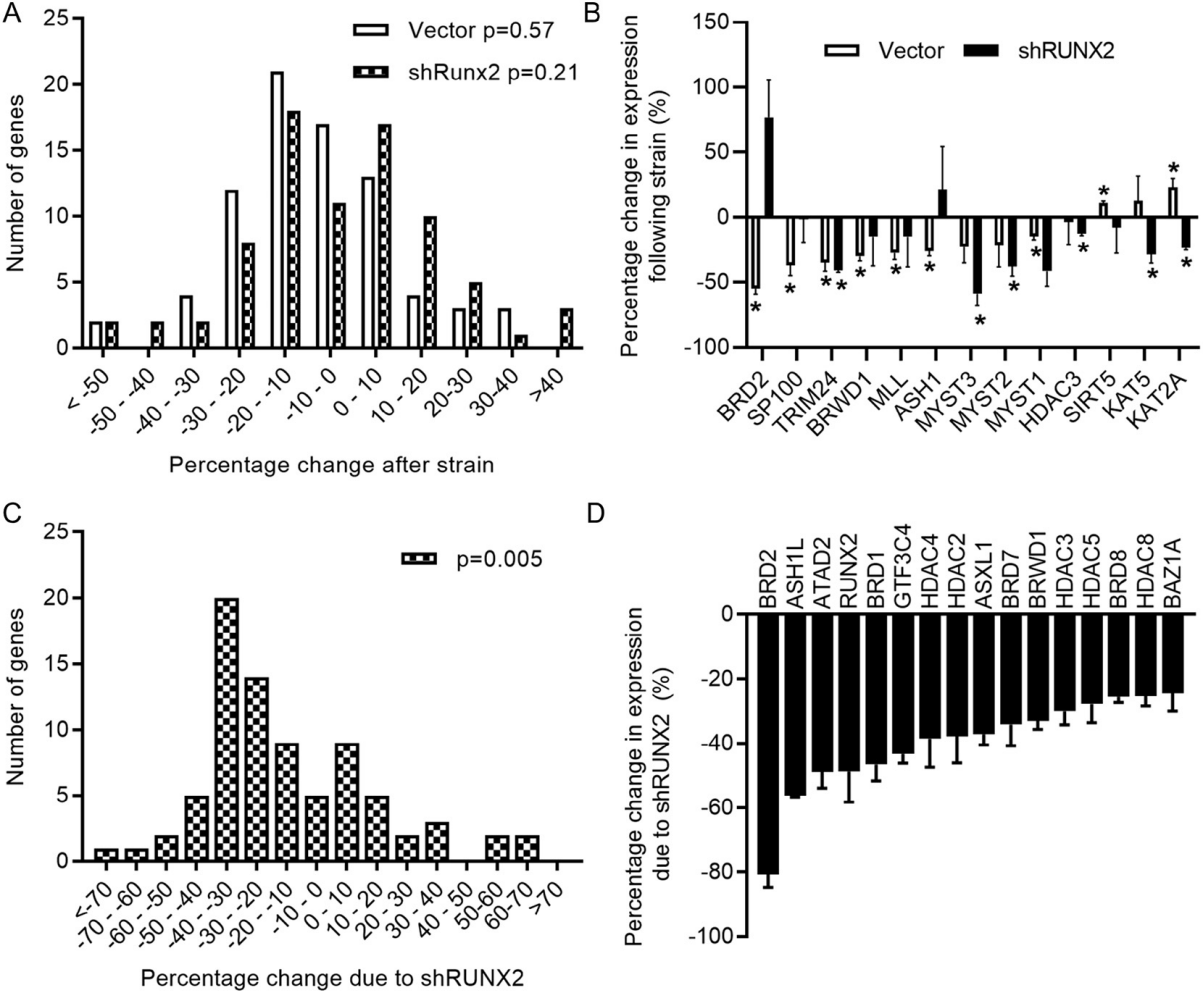
BRD2 is a strain and RUNX2 responsive gene. shRUNX2 and vector control cells were subjected to strain and harvested 8 h later for quantification of candidate epigenetic regulators using custom RT-qPCR panels. Distribution of differences in the expression of candidate epigenetic regulators in vector control or shRUNX2 (A) and shRUNX2 cells versus vector control cells (B). *P* values indicated are Shapiro-Wilk's tests for normality, indicating that RUNX2 knockdown, but not strain, skewed the distribution of percentage changes in epigenetic candidate gene expression towards down-regulation (A and B). Epigenetic regulators significantly modulated by strain (C). When genes were significantly regulated in one but not the other cell type (control or shRUNX2), the percentage changes are shown but without asterisks over the non-significant bar (* p < .05, ** p < .001 versus static controls of the same cell type). Epigenetic regulators differentially expressed between shRUNX2 and vector cells (p < .05) (B). Arrows indicate genes previously reported to be RUNX2 targets in Saos-2 cells. Bars represent the mean ± SEM, n = 3 representing three independent experiments.

RUNX2 is well known to interact with numerous epigenetic regulators which can influence promoter activity without directly altering RUNX2 expression (Jensen et al., 2007; Lamour et al., 2007; Shimizu et al., 2010). To investigate the potential implication of epigenetic regulators implicated with acetylation and methylation of histones (i.e., histone code writers, readers, and erasers), we used a custom-made PCR panel of 83 distinct genes of which 76 were robustly detected in all three repeat experiment using three housekeeping controls (HPRT, GAPDH and β2MG). Exposure to strain altered the expression of genes in our PCR panel following a normal distribution in both vector control of shRUNX2 cells (Fig. 5A). Expression analysis of epigenetic regulators (Supplementary Table 1) revealed thirteen strain-responsive genes (Fig. 5B). The bromo-domain acetyl reader BRD2 was the gene most significantly down-regulated in the vector control cells, whereas its expression was non-significantly up-regulated following strain in the shRUNX2 cells. We have previously reported strain-related regulation of Brd2 in the tibiae of both young and old mice subjected to osteogenic axial loading in vivo (Galea et al., 2017). Unlike strain, knockdown of RUNX2 resulted in a skewed distribution towards down-regulation of the investigated epigenetic regulators (Fig. 5C). Of the examined epigenetic regulators (Supplementary Table 2), all significantly regulated genes are down-regulated by RUNX2 knockdown (Fig. 5D). In addition to RUNX2 itself, the three genes most down-regulated by RUNX2 knockdown have all previously been reported to be direct RUNX2 target genes in Saos-2 cells (van der Deen et al., 2012). Altogether, these results establish that BRD2 expression is regulated by strain and RUNX2 activity.

### 3.6 BRD2 occupies the RANKL promoter but its occupancy decreases following strain

**Fig. 6:**
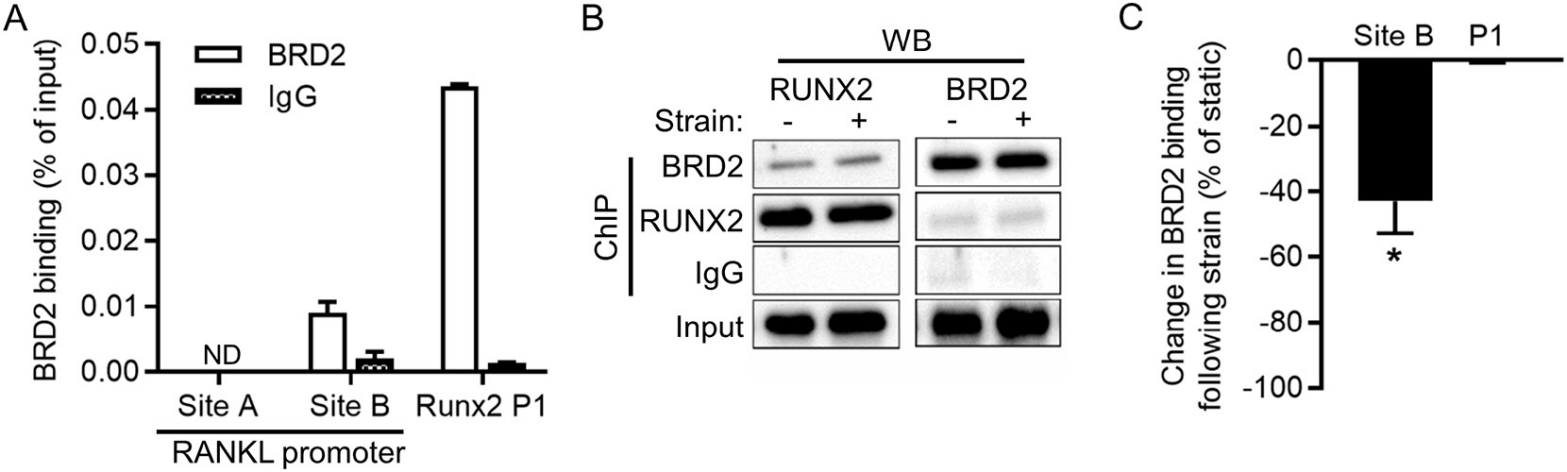
BRD2 binding to the RANKL promoter is down-regulated by strain. Saos-2 were subjected to strain and harvested 8 h later for ChIP analysis using a BRD2 and IgG antibodies. Quantification of ChIP precipitates with primers for the RANKL promoter sites A and B or the RUNX2 P1 promoter (ND = not detected) (A). Western blot analysis of ChIP lysates or Input loading control (B). Percentage change in BRD2 occupancy of the RANKL Site B and RUNX2 P1 (C). Bars represent the mean ± SEM, n = 3 representing three independent experiments. *p < .05 versus static control.

It has been established that RUNX2 occupies the BRD2 promoter in Saos-2 cells (van der Deen et al., 2012). Interestingly, BRD2 was also shown to bind to the RUNX2 promoter (Lamoureux et al., 2014). Thus, a feedback loop may exist between RUNX2 and the epigenetic reader BRD2. Similar to previous studies, ChIP analysis established BRD2 binding at the RUNX2 promoter (Fig. 6A). This assay also identified BRD2 occupancy at the RANKL promoter Site B. Western blotting of ChIP lysates demonstrated co-precipitation of RUNX2 and BRD2, suggesting they occupy the same protein complexes in both static and strained samples (Fig. 6B). Strain selectively reduced BRD2 occupancy of the RANKL promoter Site B without significantly altering its occupancy of the RUNX2 P1 promoter (Fig. 6C). These data confirm BRD2 occupancy at the RUNX2 promoter and strain-dependent occupancy at the RANKL promoter.

## 4 Discussion

The absence of bone formation in mice lacking RUNX2 demonstrates its critical role in osteoblast differentiation (Ducy et al., 1997), yet its functions in mature osteoblast lineage cells are poorly understood. Here we demonstrate that RUNX2 suppresses basal SOST expression as its knockdown increases SOST expression, suggesting RUNX2 influences the osteogenic context through sclerostin. However, RUNX2 does not mediate the acute responses to strain which result in SOST down-regulation. Conversely, RUNX2 knockdown does not alter basal RANKL expression but prevents its down-regulation by strain. In investigating potential epigenetic mechanisms by which RUNX2 mediates strain-related RANKL down-regulation, we identified an epigenetic feedback loop between RUNX2 and BRD2, demonstrating that BRD2 also occupies the RANKL promoter and that its occupancy also decreases following strain.

Epigenetic regulation of SOST expression through DNA methylation has previously been reported (Delgado-Calle et al., 2012; Reppe et al., 2015; Lhaneche et al., 2016; Stegen et al., 2018). In the present study, the up-regulation of SOST expression induced by demethylation was sub-maximal in cells lacking maximal RUNX2 expression. This is consistent with the previous report that mutation of a RUNX2 binding site in a proximal fragment of the SOST promoter reduces promoter activity (Sevetson et al., 2004). Conversely, the finding that RUNX2 knockdown increases SOST expression is consistent with the report that transfecting additional RUNX2 into Saos-2 cells decreases SOST promoter activity (Byon et al., 2011). In our model of confluent Saos-2 cells expressing a mature osteoblastic phenotype (Byon et al., 2011; Galea et al., 2013; Prideaux et al., 2014), exposure to strain did not alter RUNX2 expression while strain has been reported to up-regulate RUNX2 in marrow stromal cells which then differentiate into osteoblasts (Koike et al., 2005; Friedl et al., 2007; Kitazawa et al., 2008). RUNX2 knockdown was sufficient to reduce ALP and increase basal SOST expression, but had no effect on SOST down-regulation by strain. SOST down-regulation requires new RNA synthesis, potentially including components of the prostaglandin (Galea et al., 2011), estrogen receptor (Galea et al., 2013), nitric oxide (Delgado-Calle et al., 2014), and periostin (Bonnet et al., 2009) signaling pathways. The lack of change in basal SOST levels following 8 h of actinomycin D treatment suggest its RNA is relatively stable, at least as compared with RANKL expression which was significantly down-regulated by the same treatment. Thus, it is possible that the pathways involved in SOST down-regulation by strain may involve alterations in RNA stability, including microRNA mediated processes (Hassan et al., 2012; Taipaleenmaki et al., 2016; Qin et al., 2017; Li et al., 2019).

In contrast to its effects on SOST, knockdown of RUNX2 had no effect on basal RANKL expression. This is potentially consistent with the finding that mutating sites in the mouse RANKL promoter occupied by RUNX2 has no effect on its basal activity (O'Brien et al., 2002). RUNX2 knockdown did not alter RANKL up-regulation by PGE2. However, RUNX2 is required for strain to down-regulate RANKL in our model, suggesting that it is the presence of RUNX2 in mature osteoblastic cells which allows them to reduce the drive for resorption by down-regulating RANKL. In investigating the mechanisms by which RUNX2 mediates this, we first confirmed that RUNX2 binds the RANKL promoter in human Saos-2 cells. The RANKL promoter has been extensively studied and involves a large number of different regions and enhancer elements of varying importance in different cell types (Kitazawa et al., 1999; Bishop et al., 2011; Byon et al., 2011; Park et al., 2011; Yoskovitz et al., 2013). Here we demonstrate that a proximal region of this promoter is responsive to strain because strain decreases its occupancy by RUNX2 and BRD2.

The action of RUNX2 on target promoters is entirely dependent on its recruitment of co-activators and co-repressors such as HDACs (Lian et al., 2006; Jonason et al., 2009). We therefore used a custom PCR panel of epigenetic regulators to explore potential mechanisms by which RUNX2 occupancy of the RANKL promoter may decrease following strain. This panel reinforces the critical role RUNX2 plays in osteoblastic cell epigenetic networks, which is of recognized importance during osteoblast differentiation (Wu et al., 2014), as RUNX2 knockdown skewed the distribution of epigenetic gene expression towards down-regulation. shRUNX2 down-regulated the expression of several HDACs namely HDAC1, HDAC3 (which was further significantly down-regulated by strain in shRUNX2 but not vector control cells), HDAC4, HDAC5 and HDAC8. It has been established that HDACs bind to and modulate RUNX2 activity (Bradley et al., 2011; Bradley et al., 2015). Interestingly, our current studies suggest that RUNX2 may also govern expression of several HDACs in Saos-2 cells. The gene most significantly down-regulated by shRUNX2 was the bromodomain acetyl reader BRD2. This gene is also mechano-responsive in vivo (Galea et al., 2017).

Previously-reported ChIP-Seq experiments had identified RUNX2 occupancy of the BRD2 promoter in Saos-2 cells (van der Deen et al., 2012). It was also reported that BRD2 binds the RUNX2 promoter (Lamoureux et al., 2014), as was also observed in this study. Furthermore we report that BRD2 and RUNX2 associate within complexes. Preliminary data suggests female transgenic mice with low BRD2 expression have lower RUNX2 expression than wild type controls and develop lower bone mass as adults (Bragdon et al., 2013). Thus, these findings delineate a novel feedback loop in which RUNX2 and BRD2 occupy each other's promoters, promote each other's expression, interact within protein complexes, and occupy the same sites on target promoters potentially relevant to bone (re)modeling.

BRD2 occupancy of the RANKL promoter was significantly reduced following exposure to strain. In contrast, strain did not alter BRD2 or RUNX2 binding to the P1 promoter of RUNX2 itself, which did not show significant changes in expression following strain. This suggests that additional, as yet unknown, regulatory steps specifically target RUNX2 and BRD2 on this promoter. The proposed role of BRD2 in RANKL regulation is potentially consistent with the recently-reported findings that pharmacological inhibition of BRD proteins is associated with reduced resorption (Lamoureux et al., 2014; Meng et al., 2014; Baud'huin et al., 2017; Guo et al., 2019). Pharmacological inhibitors of BRD proteins have gained attention in the treatment of various malignancies (Xu and Vakoc, 2017). Application of these pharmacological agents, as well as studies in BRD2 low-expression mice, may be able to extend the in vitro findings presented here in future experiments.

In conclusion, RUNX2 expression in osteoblastic cells expressing SOST and RANKL influences the osteogenic context in which strain acts by suppressing basal SOST levels and contributes to the acute down-regulation of RANKL following exposure to increased strain. These effects involve reduced binding of RUNX2 to the RANKL promoter following strain. RUNX2 knockdown alters the epigenetic landscape by predominantly down-regulating epigenetic regulators normally expressed in Saos-2 cells, including BRD2. BRD2 forms a feedback loop with RUNX2 and occupies the RANKL promoter, but its occupancy is also decreased following strain. Thus, RUNX2-targeting therapies may alter bone mass through their actions on SOST and RANKL in mature osteoblastic cells. BRD2 modulators already in clinical development are also expected to have effects on bone in part through interaction with loading-related RANKL regulation.

The following are the supplementary data related to this article.

Supplementary Table 1 Effect of strain on all epigenetic regulators included in the custom PCR panel. Vector control or shRUNX2 cells were subjected to strain or kept as static controls and harvested 8 h later. The lists are sorted according to the *p* value for the effect of strain in vector control cells. Red cells indicate *p* < .05, green indicates 0.1 > *p* > .05. Data is presented as the average percentage change in expression (calculated as [strain − static] / static ∗ 100) and SEM, *n* = 3 representing three independent repeat experiments. Supplementary Table 1

Supplementary Table 2 Effect of RUNX2 knockdown on all epigenetic regulators included in the custom PCR panel. Static vector control or shRUNX2 cells were compared. The lists are sorted according to the p value associated with the percentage change in gene expression due to shRUNX2 ([shRUNX2 − vector]/vector ∗ 100). Red cells indicate p < .05, green indicates 0.1 > p > .05. Data is presented as the average and SEM, n = 3 representing three independent repeat experiments. Supplementary Table 2

## Funding

This publication was made possible by a 10.13039/100010269Wellcome Postdoctoral Clinical Research Training Fellowship (GLG), the 10.13039/100013541European Calcified Tissue Society (GLG), a 10.13039/100010269Wellcome Integrated Training Fellowships for Veterinarians (LBM), a 10.13039/100000871Mayo Clinic Career Development Award in Orthopedics Research (AD), and the 10.13039/100000069National Institute of Arthritis and Musculoskeletal and Skin Diseases (R01 AR069049 to AvW). We also thank William and Karen Eby for generous philanthropic support.

## Declaration of competing interest

The authors declare that they have no known competing financial interests or personal relationships that could have appeared to influence the work reported in this paper.

## References

[bb0005] Arbon K.S., Christensen C.M., Harvey W.A., Heggland S.J. (2012). Cadmium exposure activates the ERK signaling pathway leading to altered osteoblast gene expression and apoptotic death in Saos-2 cells. Food Chem. Toxicol..

[bb0010] Baud'huin M., Lamoureux F., Jacques C., Rodriguez Calleja L., Quillard T., Charrier C., Amiaud J., Berreur M., Brounais-LeRoyer B., Owen R., Reilly G.C., Bradner J.E., Heymann D., Ory B. (2017). Inhibition of BET proteins and epigenetic signaling as a potential treatment for osteoporosis. Bone.

[bb0015] van Bezooijen R.L., Roelen B.A.J., Visser A., van der Wee-Pals L., de Wilt E., Karperien M., Hamersma H., Papapoulos S.E., ten Dijke P., Lowik C.W.G.M. (2004). Sclerostin is an osteocyte-expressed negative regulator of bone formation, but not a classical BMP antagonist. J. Exp. Med..

[bb0020] Bishop K.A., Coy H.M., Nerenz R.D., Meyer M.B., Pike J.W. (2011). Mouse Rankl expression is regulated in T cells by c-Fos through a cluster of distal regulatory enhancers designated the T cell control region. J. Biol. Chem..

[bb0025] Blackwell K.A., Raisz L.G., Pilbeam C.C. (2010). Prostaglandins in bone: bad cop, good cop?. Trends Endocrinol. Metab..

[bb0030] Bonnet N., Standley K.N., Bianchi E.N., Stadelmann V., Foti M., Conway S.J., Ferrari S.L. (2009). The matricellular protein periostin is required for sost inhibition and the anabolic response to mechanical loading and physical activity. J. Biol. Chem..

[bb0035] Bradley E.W., McGee-Lawrence M.E., Westendorf J.J. (2011). Hdac-mediated control of endochondral and intramembranous ossification. Crit. Rev. Eukaryot. Gene Expr..

[bb0040] Bradley E.W., Carpio L.R., van Wijnen A.J., McGee-Lawrence M.E., Westendorf J.J. (2015). Histone Deacetylases in bone development and skeletal disorders. Physiol. Rev..

[bb0045] Bragdon B., Burns R., Baker A.H., Belkina A., Denis G., Morgan E.F., Gerstenfeld L.C., Schlezinger J.J. (2013). Sex-linked bone loss and adipocyte differentiation are co-regulated by the brd2 gene. 59th Annual Meeting of the Orthopaedic Research Society.

[bb0050] Byon C.H., Sun Y., Chen J., Yuan K., Mao X., Heath J.M., Anderson P.G., Tintut Y., Demer L.L., Wang D., Chen Y. (2011). Runx2-upregulated receptor activator of nuclear factor kappaB ligand in calcifying smooth muscle cells promotes migration and osteoclastic differentiation of macrophages. Arterioscler. Thromb. Vasc. Biol..

[bb0055] Compston J.E., McClung M.R., Leslie W.D. (2019). Osteoporosis. Lancet.

[bb0060] van der Deen M., Akech J., Lapointe D., Gupta S., Young D.W., Montecino M.A., Galindo M., Lian J.B., Stein J.L., Stein G.S., van Wijnen A.J. (2012). Genomic promoter occupancy of runt-related transcription factor RUNX2 in osteosarcoma cells identifies genes involved in cell adhesion and motility. J. Biol. Chem..

[bb0065] Delgado-Calle J., Sanudo C., Bolado A., Fernandez A.F., Arozamena J., Pascual-Carra M.A., Rodriguez-Rey J.C., Fraga M.F., Bonewald L., Riancho J.A. (2012). DNA methylation contributes to the regulation of sclerostin expression in human osteocytes. J. Bone Miner. Res..

[bb0070] Delgado-Calle J., Riancho J.A., Klein-Nulend J. (2014). Nitric oxide is involved in the down-regulation of SOST expression induced by mechanical loading. Calcif. Tissue Int..

[bb0075] Ducy P., Zhang R., Geoffroy V., Ridall A.L., Karsenty G. (1997). Osf2/Cbfa1: a transcriptional activator of osteoblast differentiation. Cell.

[bb0080] Dudakovic A., Evans J.M., Li Y., Middha S., McGee-Lawrence M.E., van Wijnen A.J., Westendorf J.J. (2013). Histone deacetylase inhibition promotes osteoblast maturation by altering the histone H4 epigenome and reduces Akt phosphorylation. J. Biol. Chem..

[bb0085] Dudakovic A., Camilleri E.T., Riester S.M., Paradise C.R., Gluscevic M., O’Toole T.M., Thaler R., Evans J.M., Yan H., Subramaniam M., Hawse J.R., Stein G.S., Montecino M.A., McGee-Lawrence M.E., Westendorf J.J., van Wijnen A.J. (2016). Enhancer of Zeste homolog 2 inhibition stimulates bone formation and mitigates bone loss caused by Ovariectomy in skeletally mature mice. J. Biol. Chem..

[bb0090] Dudakovic A., Gluscevic M., Paradise C.R., Dudakovic H., Khani F., Thaler R., Ahmed F.S., Li X., Dietz A.B., Stein G.S., Montecino M.A., Deyle D.R., Westendorf J.J., van Wijnen A.J. (2017). Profiling of human epigenetic regulators using a semi-automated real-time qPCR platform validated by next generation sequencing. Gene.

[bb0095] Fan X., Rahnert J.A., Murphy T.C., Nanes M.S., Greenfield E.M., Rubin J. (2006). Response to mechanical strain in an immortalized pre-osteoblast cell is dependent on ERK1/2. J. Cell. Physiol..

[bb0100] Feher A., Koivunemi A., Koivunemi M., Fuchs R.K., Burr D.B., Phipps R.J., Reinwald S., Allen M.R. (2010). Bisphosphonates do not inhibit periosteal bone formation in estrogen deficient animals and allow enhanced bone modeling in response to mechanical loading. Bone.

[bb0105] Friedl G., Schmidt H., Rehak I., Kostner G., Schauenstein K., Windhager R. (2007). Undifferentiated human mesenchymal stem cells (hMSCs) are highly sensitive to mechanical strain: transcriptionally controlled early osteo-chondrogenic response in vitro. Osteoarthr. Cartil..

[bb0110] Galea G.L., Sunters A., Meakin L.B., Zaman G., Sugiyama T., Lanyon L.E., Price J.S. (2011). Sost down-regulation by mechanical strain in human osteoblastic cells involves PGE2 signaling via EP4. FEBS Lett..

[bb0115] Galea G.L., Meakin L.B., Sugiyama T., Zebda N., Sunters A., Taipaleenmaki H., Stein G.S., van Wijnen A.J., Lanyon L.E., Price J.S. (2013). Estrogen receptor alpha mediates proliferation of osteoblastic cells stimulated by estrogen and mechanical strain, but their acute down-regulation of the Wnt antagonist Sost is mediated by estrogen receptor beta. J. Biol. Chem..

[bb0120] Galea G.L., Meakin L.B., Williams C.M., Hulin-Curtis S.L., Lanyon L.E., Poole A.W., Price J.S. (2014). Protein kinase Calpha (PKCalpha) regulates bone architecture and osteoblast activity. J. Biol. Chem..

[bb0125] Galea G.L., Meakin L.B., Savery D., Taipaleenmaki H., Delisser P., Stein G.S., Copp A.J., van Wijnen A.J., Lanyon L.E., Price J.S. (2015). Planar cell polarity aligns osteoblast division in response to substrate strain. J. Bone Miner. Res..

[bb0130] Galea G.L., Meakin L.B., Harris M.A., Delisser P.J., Lanyon L.E., Harris S.E., Price J.S. (2017). Old age and the associated impairment of bones' adaptation to loading are associated with transcriptomic changes in cellular metabolism, cell-matrix interactions and the cell cycle. Gene.

[bb0135] Gaudio A., Pennisi P., Bratengeier C., Torrisi V., Lindner B., Mangiafico R.A., Pulvirenti I., Hawa G., Tringali G., Fiore C.E. (2010). Increased sclerostin serum levels associated with bone formation and resorption markers in patients with immobilization-induced bone loss. J. Clin. Endocrinol. Metab..

[bb0140] Genetos D.C., Yellowley C.E., Loots G.G. (2011). Prostaglandin E2 signals through PTGER2 to regulate sclerostin expression. PLoS One.

[bb0145] Guo N.H., Zheng J.F., Zi F.M., Cheng J. (2019). I-BET151 suppresses osteoclast formation and inflammatory cytokines secretion by targetting BRD4 in multiple myeloma. Biosci. Rep..

[bb0150] Hassan M.Q., Maeda Y., Taipaleenmaki H., Zhang W., Jafferji M., Gordon J.A., Li Z., Croce C.M., van Wijnen A.J., Stein J.L., Stein G.S., Lian J.B. (2012). miR-218 directs a Wnt signaling circuit to promote differentiation of osteoblasts and Osteomimicry of metastatic cancer cells. J. Biol. Chem..

[bb0155] Holmen S.L., Zylstra C.R., Mukherjee A., Sigler R.E., Faugere M.C., Bouxsein M.L., Deng L., Clemens T.L., Williams B.O. (2005). Essential role of beta-catenin in postnatal bone acquisition. J. Biol. Chem..

[bb0160] Jensen E.D., Nair A.K., Westendorf J.J. (2007). Histone deacetylase co-repressor complex control of Runx2 and bone formation. Crit. Rev. Eukaryot. Gene Expr..

[bb0165] Jonason J.H., Xiao G., Zhang M., Xing L., Chen D. (2009). Post-translational regulation of Runx2 in bone and cartilage. J. Dent. Res..

[bb0170] Kanno T., Takahashi T., Tsujisawa T., Ariyoshi W., Nishihara T. (2007). Mechanical stress-mediated Runx2 activation is dependent on Ras/ERK1/2 MAPK signaling in osteoblasts. J. Cell. Biochem..

[bb0175] Kennedy O.D., Herman B.C., Laudier D.M., Majeska R.J., Sun H.B., Schaffler M.B. (2012). Activation of resorption in fatigue-loaded bone involves both apoptosis and active pro-osteoclastogenic signaling by distinct osteocyte populations. Bone.

[bb0180] Kitazawa R., Kitazawa S., Maeda S. (1999). Promoter structure of mouse RANKL/TRANCE/OPGL/ODF gene. Biochim. Biophys. Acta.

[bb0185] Kitazawa R., Mori K., Yamaguchi A., Kondo T., Kitazawa S. (2008). Modulation of mouse RANKL gene expression by Runx2 and vitamin D3. J. Cell. Biochem..

[bb0190] Kitazawa S., Kajimoto K., Kondo T., Kitazawa R. (2003). Vitamin D3 supports osteoclastogenesis via functional vitamin D response element of human RANKL gene promoter. J. Cell. Biochem..

[bb0195] Koike M., Shimokawa H., Kanno Z., Ohya K., Soma K. (2005). Effects of mechanical strain on proliferation and differentiation of bone marrow stromal cell line ST2. J. Bone Miner. Metab..

[bb0200] Kusumi A., Sakaki H., Kusumi T., Oda M., Narita K., Nakagawa H., Kubota K., Satoh H., Kimura H. (2005). Regulation of synthesis of osteoprotegerin and soluble receptor activator of nuclear factor-kappaB ligand in normal human osteoblasts via the p38 mitogen-activated protein kinase pathway by the application of cyclic tensile strain. J. Bone Miner. Metab..

[bb0205] Kusumi A., Kusumi T., Miura J., Tateishi T. (2009). Passage-affected competitive regulation of osteoprotegerin synthesis and the receptor activator of nuclear factor-kappaB ligand mRNA expression in normal human osteoblasts stimulated by the application of cyclic tensile strain. J. Bone Miner. Metab..

[bb0210] Lamour V., Detry C., Sanchez C., Henrotin Y., Castronovo V., Bellahcene A. (2007). Runx2- and histone deacetylase 3-mediated repression is relieved in differentiating human osteoblast cells to allow high bone sialoprotein expression. J. Biol. Chem..

[bb0215] Lamoureux F., Baud’huin M., Rodriguez Calleja L., Jacques C., Berreur M., Redini F., Lecanda F., Bradner J.E., Heymann D., Ory B. (2014). Selective inhibition of BET bromodomain epigenetic signalling interferes with the bone-associated tumour vicious cycle. Nat. Commun..

[bb0220] Lhaneche L., Hald J.D., Domingues A., Hannouche D., Delepine M., Zelenika D., Boland A., Ostertag A., Cohen-Solal M., Langdahl B.L., Harslof T., de Vernejoul M.C., Geoffroy V., Jehan F. (2016). Variations of SOST mRNA expression in human bone are associated with DNA polymorphism and DNA methylation in the SOST gene. Bone.

[bb0225] Li L., Qiu X., Sun Y., Zhang N., Wang L. (2019). SP1-stimulated miR-545-3p inhibits osteogenesis via targeting LRP5-activated Wnt/beta-catenin signaling. Biochem. Biophys. Res. Commun..

[bb0230] Lian J.B., Stein G.S., Javed A., van Wijnen A.J., Stein J.L., Montecino M., Hassan M.Q., Gaur T., Lengner C.J., Young D.W. (2006). Networks and hubs for the transcriptional control of osteoblastogenesis. Rev. Endocr. Metab. Disord..

[bb0235] McGee-Lawrence M.E., McCleary-Wheeler A.L., Secreto F.J., Razidlo D.F., Zhang M., Stensgard B.A., Li X., Stein G.S., Lian J.B., Westendorf J.J. (2011). Suberoylanilide hydroxamic acid (SAHA; vorinostat) causes bone loss by inhibiting immature osteoblasts. Bone.

[bb0240] Meakin L.B., Galea G.L., Sugiyama T., Lanyon L.E., Price J.S. (2014). Age-related impairment of Bones’ adaptive response to loading in mice is associated with gender-related deficiencies in osteoblasts but no change in osteocytes. J. Bone Miner. Res..

[bb0245] Meng S., Zhang L., Tang Y., Tu Q., Zheng L., Yu L., Murray D., Cheng J., Kim S.H., Zhou X., Chen J. (2014). BET inhibitor JQ1 blocks inflammation and bone destruction. J. Dent. Res..

[bb0250] Moriishi T., Fukuyama R., Ito M., Miyazaki T., Maeno T., Kawai Y., Komori H., Komori T. (2012). Osteocyte network; a negative regulatory system for bone mass augmented by the induction of Rankl in osteoblasts and Sost in osteocytes at unloading. PLoS One.

[bb0255] Moustafa A., Sugiyama T., Prasad J., Zaman G., Gross T.S., Lanyon L.E., Price J.S. (2012). Mechanical loading-related changes in osteocyte sclerostin expression in mice are more closely associated with the subsequent osteogenic response than the peak strains engendered. Osteoporos Int Apr.

[bb0260] O’Brien C.A., Kern B., Gubrij I., Karsenty G., Manolagas S.C. (2002). Cbfa1 does not regulate RANKL gene activity in stromal/osteoblastic cells. Bone.

[bb0265] Park H.J., Baek K.H., Lee H.L., Kwon A., Hwang H.R., Qadir A.S., Woo K.M., Ryoo H.M., Baek J.H. (2011). Hypoxia inducible factor-1alpha directly induces the expression of receptor activator of nuclear factor-kappaB ligand in periodontal ligament fibroblasts. Mol Cells.

[bb0270] Pichler K., Loreto C., Leonardi R., Reuber T., Weinberg A.M., Musumeci G. (2013). RANKL is downregulated in bone cells by physical activity (treadmill and vibration stimulation training) in rat with glucocorticoid-induced osteoporosis. Histol. Histopathol..

[bb0275] Pratap J., Wixted J.J., Gaur T., Zaidi S.K., Dobson J., Gokul K.D., Hussain S., van Wijnen A.J., Stein J.L., Stein G.S., Lian J.B. (2008). Runx2 transcriptional activation of Indian hedgehog and a downstream bone metastatic pathway in breast cancer cells. Cancer Res..

[bb0280] Prideaux M., Wijenayaka A.R., Kumarasinghe D.D., Ormsby R.T., Evdokiou A., Findlay D.M., Atkins G.J. (2014). SaOS2 osteosarcoma cells as an in vitro model for studying the transition of human osteoblasts to osteocytes. Calcif. Tissue Int..

[bb0285] Qin Y., Peng Y., Zhao W., Pan J., Ksiezak-Reding H., Cardozo C., Wu Y., Divieti Pajevic P., Bonewald L.F., Bauman W.A., Qin W. (2017). Myostatin inhibits osteoblastic differentiation by suppressing osteocyte-derived exosomal microRNA-218: a novel mechanism in muscle-bone communication. J. Biol. Chem..

[bb0290] Rahnert J., Fan X., Case N., Murphy T.C., Grassi F., Sen B., Rubin J. (2008). The role of nitric oxide in the mechanical repression of RANKL in bone stromal cells. Bone.

[bb0295] Reppe S., Noer A., Grimholt R.M., Halldorsson B.V., Medina-Gomez C., Gautvik V.T., Olstad O.K., Berg J.P., Datta H., Estrada K., Hofman A., Uitterlinden A.G., Rivadeneira F., Lyle R., Collas P., Gautvik K.M. (2015). Methylation of bone SOST, its mRNA, and serum sclerostin levels correlate strongly with fracture risk in postmenopausal women. J. Bone Miner. Res..

[bb0300] Robling A.G., Niziolek P.J., Baldridge L.A., Condon K.W., Allen M.R., Alam I., Mantila S.M., Gluhak-Heinrich J., Bellido T.M., Harris S.E., Turner C.H. (2008). Mechanical stimulation of bone in vivo reduces osteocyte expression of Sost/sclerostin. J. Biol. Chem..

[bb0305] Romagnoli C., Marcucci G., Favilli F., Zonefrati R., Mavilia C., Galli G., Tanini A., Iantomasi T., Brandi M.L., Vincenzini M.T. (2013). Role of GSH/GSSG redox couple in osteogenic activity and osteoclastogenic markers of human osteoblast-like SaOS-2 cells. FEBS J..

[bb0310] Rubin J., Murphy T., Nanes M.S., Fan X. (2000). Mechanical strain inhibits expression of osteoclast differentiation factor by murine stromal cells. Am J Physiol Cell Physiol.

[bb0315] Rubin J., Murphy T.C., Zhu L., Roy E., Nanes M.S., Fan X. (2003). Mechanical strain differentially regulates endothelial nitric-oxide synthase and receptor activator of nuclear kappa B ligand expression via ERK1/2 MAPK. J. Biol. Chem..

[bb0320] Rucci N., Rufo A., Alamanou M., Teti A. (2007). Modeled microgravity stimulates osteoclastogenesis and bone resorption by increasing osteoblast RANKL/OPG ratio. J. Cell. Biochem..

[bb0325] Sevetson B., Taylor S., Pan Y. (2004). Cbfa1/RUNX2 directs specific expression of the sclerosteosis gene (SOST). J. Biol. Chem..

[bb0330] Shimizu E., Selvamurugan N., Westendorf J.J., Olson E.N., Partridge N.C. (2010). HDAC4 represses matrix metalloproteinase-13 transcription in osteoblastic cells, and parathyroid hormone controls this repression. J. Biol. Chem..

[bb0335] Sims N.A., Martin T.J. (2014). Coupling the activities of bone formation and resorption: a multitude of signals within the basic multicellular unit. Bonekey Rep.

[bb0340] Spandidos A., Wang X., Wang H., Seed B. (2010). PrimerBank: a resource of human and mouse PCR primer pairs for gene expression detection and quantification. Nucleic Acids Res..

[bb0345] Stegen S., Stockmans I., Moermans K., Thienpont B., Maxwell P.H., Carmeliet P., Carmeliet G. (2018). Osteocytic oxygen sensing controls bone mass through epigenetic regulation of sclerostin. Nat. Commun..

[bb0350] Taipaleenmaki H., Farina N.H., van Wijnen A.J., Stein J.L., Hesse E., Stein G.S., Lian J.B. (2016). Antagonizing miR-218-5p attenuates Wnt signaling and reduces metastatic bone disease of triple negative breast cancer cells. Oncotarget.

[bb0355] Tatsumi S., Ishii K., Amizuka N., Li M., Kobayashi T., Kohno K., Ito M., Takeshita S., Ikeda K. (2007). Targeted ablation of osteocytes induces osteoporosis with defective mechanotransduction. Cell Metab..

[bb0360] Tsutsumi R., Xie C., Wei X., Zhang M., Zhang X., Flick L.M., Schwarz E.M., O’Keefe R.J. (2009). PGE2 signaling through the EP4 receptor on fibroblasts upregulates RANKL and stimulates osteolysis. J. Bone Miner. Res..

[bb0365] Wu H., Whitfield T.W., Gordon J.A., Dobson J.R., Tai P.W., van Wijnen A.J., Stein J.L., Stein G.S., Lian J.B. (2014). Genomic occupancy of Runx2 with global expression profiling identifies a novel dimension to control of osteoblastogenesis. Genome Biol..

[bb0370] Xiong J., Onal M., Jilka R.L., Weinstein R.S., Manolagas S.C., O’Brien C.A. (2011). Matrix-embedded cells control osteoclast formation. Nat. Med..

[bb0375] Xu Y., Vakoc C.R. (2017). Targeting cancer cells with BET Bromodomain inhibitors. Cold Spring Harb Perspect Med.

[bb0380] Yasuda H., Shima N., Nakagawa N., Yamaguchi K., Kinosaki M., Mochizuki S., Tomoyasu A., Yano K., Goto M., Murakami A., Tsuda E., Morinaga T., Higashio K., Udagawa N., Takahashi N., Suda T. (1998). Osteoclast differentiation factor is a ligand for osteoprotegerin/osteoclastogenesis-inhibitory factor and is identical to TRANCE/RANKL. Proc. Natl. Acad. Sci. U. S. A..

[bb0385] Yoskovitz G., Garcia-Giralt N., Rodriguez-Sanz M., Urreizti R., Guerri R., Arino-Ballester S., Prieto-Alhambra D., Mellibovsky L., Grinberg D., Nogues X., Balcells S., Diez-Perez A. (2013). Analyses of RANK and RANKL in the post-GWAS context: functional evidence of vitamin D stimulation through a RANKL distal region. J. Bone Miner. Res..

